# High‐throughput analysis of anammox bacteria in wetland and dryland soils along the altitudinal gradient in Qinghai–Tibet Plateau

**DOI:** 10.1002/mbo3.556

**Published:** 2017-12-29

**Authors:** Siyan Zhao, Linjie Zhuang, Cheng Wang, Yifei Li, Shanyun Wang, Guibing Zhu

**Affiliations:** ^1^ Research Center for Eco‐Environmental Sciences Chinese Academy of Sciences Beijing China; ^2^ University of Chinese Academy of Sciences Beijing China; ^3^ School of Environment and Civil Engineering Jiangnan University Wuxi China

**Keywords:** abundance, anammox bacteria, community composition, diversity, high throughput, Qinghai–Tibet Plateau

## Abstract

This study investigated the diversity, community composition, and abundance of anaerobic ammonium oxidation (anammox) bacteria along the altitudinal gradient in Qinghai–Tibet Plateau. Two types of soil samples (wetland and dryland soils, *n *=* *123) were collected from 641 m to 5,033 m altitudes. Polymerase chain reaction (PCR) screening showed that anammox were not widespread, and were only detected in 9 sampling sites of the 50 sites tested by amplifying the 16S rRNA genes. Then, only samples collected from Linzhi (2,715 m), Rikaze (4,030 m), and Naqu (5,011 m), which were positive for the presence of anammox, were further processed to explore the biogeography of anammox bacteria in Qinghai–Tibet Plateau. Results of high‐throughput sequencing targeting the hydrazine synthesis β‐subunit (*hzs*B) gene revealed the presence of three known anammox genera (*Candidatus* Brocadia, *Candidatus* Jettenia, and *Candidatus* Kuenenia) in both soil types. Their diversity, community composition, and abundance did not show significant variation with altitude at large scale. However, it was the small‐scale environmental heterogeneities between wetland and dryland soils that determined their biogeographical distribution. Specifically, the dryland soils had higher diversity of anammox bacteria than the wetland soils, but their abundance patterns varied. The community composition of anammox bacteria were found to be influenced by soil nitrate content.

## 1 INTRODUCTION

Recent knowledge generated in the last 10 years showed that anaerobic ammonium oxidation (anammox) to dinitrogen gas driven by anammox bacteria is a key process in the global nitrogen cycle (Dalsgaard, Bo, & Canfield, 2005; Engström, Dalsgaard, Hulth, & Aller, 2005; Strous & Jetten, 2004). However, due to the low growth rate, nutrient selectivity, and sensitivity of anammox bacteria (Strous, Heijnen, Kuenen, & Jetten, 1998), they have not yet been isolated as pure cultures. Although studies on highly enriched mixed cultures permitted physiological characterization of some representatives and led to the description of five genera, that is, *Candidatus* Kuenenia (Schmid et al., 2000), *Candidatus* Brocadia (Strous et al., 1999), *Candidatus* Scalindua (Damsté et al., 2003), *Candidatus* Anammoxoglobus (Kartal et al., 2007), *Candidatus* Jettenia (Quan et al., 2008), and *Candidatus* Anammoximicrobium (Khramenkov et al., 2013). Most knowledge on the diversity of these taxa were mainly based on environmental surveys of amplified 16S rRNA gene, and to date, has been the most widely used approach in detecting these uncultivable taxonomic groups (Schmid et al., 2005). However, the highly conserved nature of 16S rRNA limits its efficacy and sensitivity in examining the true diversity of anammox bacterial communities and their distribution in various environments (Hirsch, Long, & Song, 2011). Several studies have also shown that the commonly used 16S rRNA gene primers could not reveal the extent of diversity of these anammox bacteria (Bale et al., 2014; Harhangi et al., 2012; Lipsewers et al., 2014).

Recent advances in molecular methods mainly targeting functional genes have enhanced the detection and understanding of the microbial diversity involved in the nitrogen cycle (Braker, Zhou, Wu, Devol, & Tiedje, 2000; Francis, O'Mullan, & Ward, 2003; Herfort et al., 2009; Jayakumar, Francis, Naqvi, & Ward, 2004; Smith, Nedwell, Dong, & Osborn, 2007). The gene coding for hydrazine synthase (HZS) in particular, which is responsible for the synthesis of hydrazine from nitric oxide and ammonium, is proposed to be a useful genetic marker for anammox bacteria (Kartal et al., 2011). It consists of three subunits (i.e., hydrazine synthesis α, β, and γ subunits) encoded by the genes *hzs*A, *hzs*B, and *hzs*C, respectively (Harhangi et al., 2012; Kartal et al., 2011; Strous et al., 2006; Wang, Zhu, Peng, Jetten, & Yin, 2012). Among these, the *hzs*B gene has been regarded as a good biomarker to determine the diversity and abundance of anammox bacteria (Wang et al., 2012; Zhu et al., 2013, 2015), and has been widely used to study the occurrence and composition of anammox bacteria in various ecosystems (Bai, Chen, He, Shen, & Zhang, 2015; Xi, Ren, Zhang, & Fang, 2016; Yang et al., 2015; Zhu et al., 2015). In addition, the fast‐paced developments in high‐throughput sequencing make it feasible to obtain a more comprehensive understanding, especially in the field of microbial community ecology (Adetutu et al., 2012). However, the use of high‐throughput sequencing approach to profile the anammox community based on *hsz*B gene has not yet been carried out.

Anammox bacteria are ubiquitously distributed at redox transition zones in marine (Dalsgaard et al., 2005; Kuypers et al., 2003), freshwater (Schubert et al., 2006; Zhang et al., 2007), and terrestrial ecosystems (Humbert et al., 2010; Shen et al., 2013). Their distribution, community composition, and abundance in these various ecosystems are affected by numerous environmental factors, such as sediment organic C/organic N (OrgC/OrgN), nitrite concentration, and sediment median grain size (Dang et al., 2010). In Pearl Estuary, for example, patterns of distribution of anammox bacteria were associated with salinity, temperature, and pH of the overlying water mass (Fu et al., 2015). Most studies conducted on the community of these taxa, however, were based on aquatic ecosystem, and to date, only few studies are available based on terrestrial ecosystems including the alpine ecosystems (Long, Heitman, Tobias, Philips, & Song, 2013; Xi et al., 2016). These alpine ecosystems are quite unique as they receive low reactive nitrogen input, and understanding the role of the anammox bacteria in such conditions would have profound implications on the general understanding of nitrogen cycle. In particular, there is still a large gap existing in the study on anammox bacterial distribution in representative environments (wetland and dryland) of the alpine ecosystems.

Hence, the goals of this study are: (1) to explore the occurrence of anammox bacteria in alpine ecosystems and (2) to study their diversity, community composition, and abundance, and the crucial environmental factors that influence their biogeography. To achieve these, we collected samples from the Qinghai–Tibet Plateau, which is considered as the Earth's third polar region. Two representative habitats, a wetland and a dryland, situated along an altitudinal gradient were selected as the sampling sites. Community composition and diversity of anammox bacteria were determined by Illumina sequencing analysis targeting the *hzs*B gene. We then applied multivariate statistical analyses to link the community patterns with potential environmental factors influencing their biogeography in Qinghai–Tibet Plateau. Our findings contribute to the further understanding of the anammox process and the biogeography of anammox bacteria.

## 2 MATERIALS AND METHODS

### 2.1 Site description and soil sampling

The Qinghai–Tibet Plateau (26°00′–39°47′ N, 73°19′–104°47′ E) ranges from the southern Himalaya Mountains to northern Kunlun Mountains, Altun Mountains, and Qilian Mountains, bounded by the Pamir and Karakoram Mountains in the west, and Qinling Mountains and Loess Plateau in the east. It is the highest plateau on earth and known as “the roof of the world” with an average altitude of more than 4,000 m, covering 2.5 million·km^2^, nearly a quarter of the total land area of China. It is surrounded and traversed by several snow‐capped mountain ranges and is the origin of many major rivers. It is characterized by strong solar radiation (3,000–6,000 MJ/m^2^), but its temperature decreases with altitude, with an annual average temperature below 5°C, particularly −15°C to −2°C in winter and 8°C to 18°C in summer. The mean annual precipitation in the Qinghai–Tibet Plateau is 360 mm and varies significantly from the southeast to the northwest. Diverse soil types including river and lake wetlands, grassland and permafrost developed in this region along the altitude, and anthropogenic activities have rare impact on those above 5,000 m. All climatological information was obtained from China Meteorological Data Service Center (http://data.cma.cn/).

Our study was conducted along the altitude range of 4,392 m, from Ya'an (641 m) to Yang bajing (5,033 m) in Qinghai–Tibet Plateau, across different regions including Ya'an, Luding, Tongmai, Kangding, Batang, Bomi, Bayi, Linzhi, Basu, Lulang, Gongbujiangda, Qushui, Xinduqiao, Lasa, Mozhugongka, Rikaze, Mangkang, Zuogong, Renbu, Ranwu, Litang, Lazi, Naqu, Dangxiong, Bangda, Gulu, and Yangbajing, 27 regions in total (29°13′ N to 31°04′ N, 87°37′E to 103°00′ E; Table S1) were selected. Wetland and dryland are two typical ecosystems, and the characters of the soils in each ecosystem are also very different. Wetland ecosystems include marsh, swamp, mires, and aquatic ecosystems like river, lake, and stream (Keddy, 2010). But dryland is a relatively arid region with forest, grassland, and farmland as the most common types of land use. So we collected both two types of soils at each sampling region to understand the biogeography of anammox bacteria better. At each sampling site, after the surface dead plants were removed, soil samples were collected at 0–10 cm depth by using soil drill with three parallel samples. The soil samples were placed in sterile plastic bags, sealed, and transported to the laboratory with ice packs and protected from light. At each site, three parallel samples were mixed to form one composite sample. Each mixed sample was divided into two parts: one part was passed through a 2‐mm sieve for physicochemical properties analysis, and the other part was frozen at −80°C for molecular analysis.

### 2.2 Analytical procedures for soil environmental variables

The soil chemical and physical properties were measured according to Bao (2000). Briefly, soil ammonia nitrogen, nitrite nitrogen, and nitrate nitrogen concentrations were measured by the SEAL Auto Analyzer 3 HR (Seal Analytical, UK) after extraction with KCl (1:5 soil/2 mol/L KCl solution) with detection limits of 0.015 mg·kg^−1^, 0.015 mg·kg^−1^, and 0.03 mg·kg^−1^, respectively. The soil pH was measured by the DELTA 320 pH Analyzer (Mettler Toledo, USA) in a suspension of 1:5 soil:water. The soil moisture contents were analyzed by oven drying 2 g of fresh soil at 108°C until a constant weight was achieved, and then the samples were placed into a microwave muffle furnace at 550°C for 5 hr to determine the total organic matter (TOM) with detection limit of 0.02 g·kg^−1^. The total nitrogen (TN), total carbon (TC), and total sulfur (TS) concentrations were determined using a Vario EL III Analyzer (Elementar Analyses System GmbH, Germany) with detection limits of 0.05 mg·kg^−1^, 0.2 mg·kg^−1^, and 0.25 mg·kg^−1^. Triplicates were run for quality assurance/quality control (QA/QC) for all above measurements.

### 2.3 DNA extraction, polymerase chain reaction, and Illumina sequencing

About 0.33 g of freeze‐dried soil of each sample was used for DNA extraction by following the manufacturer's protocol of FastDNA SPIN Kit for Soil (MP Biomedical, Solon, OH, USA). The extracted DNA was checked on 1% agarose gel and its concentration was determined using a Nanodrop^®^ ND‐2000 ultraviolet–visible spectrophotometer (Thermo Fisher Scientific, Schwerte, Germany). The ratio of the absorbance at 260 nm and 280 nm were all about 1.8, which indicated that DNA with a good quality was obtained.

A nest PCR was conducted targeting the hydrazine synthease β‐subunit (*hzs*B) gene which is specific to anammox bacteria using the primer sets of HSB396F‐HSB742R and HSB449F (barcoded)‐HSB742R performed on a C1000 Thermal Cycler (Bio‐Rad, USA) (Wang et al., 2012). The amplification mixture (50 μl) contained 5 μl of 10× buffer, 4 μl of dNTP (2.5 mmol·L^−1^), 1 μl of each primer (10 mmol·L^−1^), 0.5 μl of BSA, 0.25 μl of Taq (2.5 U), 2 μl of DNA, and was topped up with ddH_2_O to a total volume of 50 μl. The PCR operation conditions consisted of an initial 95°C for 10 min, 35 cycles of 95°C for 60 s, 59°C for 60 s, and a final extension at 72°C for 45 s. High‐throughput paired end Illumina HiSeq sequencing (2 × 250 bp) was performed at NovoGene, Beijing, China.

### 2.4 Quantitative real‐time PCR

SYBR Green I based real‐time PCR assays were carried out in a volume of 20 μl, containing 10 μl of SYBR^®^ Premix Ex Taq^™^ (TAKARA, Dalian, China), 0.4 μl of each primer (10 pmol/μl), and 2 μl of 10‐fold diluted DNA template, and was topped up with ddH_2_O to a total volume of 20 μl. Amplification and detection were carried out using an ABI Prism 7300 Sequence Detection System (Applied Biosystems, USA) with the primer sets and thermal profiles described as above. Three no‐template controls (NTCs) were run for each quantitative PCR assay. Ten‐fold serial dilutions of a known copy number of the plasmid DNA were subjected to real‐time PCR in triplicate to generate an external standard curve. Melting curves were generated after each assay to check the specificity of amplification. PCR efficiencies were 90%–103% (average 92%) and only the results with correlation coefficient above 0.98 were employed. The melt curve analyses were performed to confirm the specificity of PCR amplifications. All tests were performed in triplicate.

The detection limit of the *hzs*B gene was determined by amplifying the diluted plasmid DNA (10‐fold dilution series) with the *hzs*B gene as insert. The sample would be assumed to be at the detection limit in three situations: (1) the last sample with *C*_t_ value showed high standard deviation between replicates; or (2) the sample with *C*_t_ value was more than that of the negative control (normally the *C*_t_ of negative control was around 40 in this study) as shown in Figure S2; or (3) the melting curve was not run as a single peak. Using the *hzs*B gene on a plasmid, detection limit was around 1.34 × 10^2^ copies judging from the amplified standard curve (Figure S1). For the environmental samples like the soils in Qinghai–Tibet Plateau which can never be as clean as the plasmid DNA sample, *hzs*B gene copies would be no longer reliable when the number was lower than 1.34 × 10^3^ copies·g^−1^. This number was the detection limit used in this environmental investigation.

### 2.5 Sequencing analysis

Sequencing reads were assigned to each sample according to the unique 6‐bp barcode of each sample. The barcode and primers then were removed. Raw sequences from original DNA fragments were merged using FLASH (Magoč and Salzberg, 2011) and then were filtered (i.e., with a quality score <25 and read length <200 bp were filtered using the split_libraries command) using the QIIME software package (Caporaso et al., 2010). Then, the chimeric sequences were removed using UCHIME (Edgar, Haas, Clemente, Quince, & Knight, 2011). To accurately detect and correct frameshifts caused by indel sequencing errors, the FrameBot (Wang et al., 2013) tool was used. Briefly, only the sequences containing no ambiguous bases (N), without any barcode or primer mismatches, and with the corrected frameshifts and length (about 292 bp) were included into the downstream analysis. The unique sequences were obtained by Mothur (Schloss et al., 2009) from the remaining high‐quality nucleic acid sequences and then translated to protein sequences. Preprocessed sequences were clustered into operational taxonomic units (OTUs) based on their sequence similarity using UCLUST (identity = 0.90) (Edgar, 2010). A representative sequence for each OTUs was finally aligned using MUSCLE program (Edgar, 2004). A local alignment search was conducted with *hzs*B protein sequences using Basic Local Alignment Search Tool (BLAST).

Singletons were excluded and resampling according to the minimum sequence numbers across all samples was performed before calculation. A variety of alpha diversity indices including Chao1, Shannon, and Simpson were calculated by Mothur software. The Chao1 measured the richness of phylotypes, Shannon index estimated for both richness and evenness, whereas Simpson index detected dominant OTUs in the samples. The beta diversity was determined by weighted and unweighted UniFrac (Lozupone & Knight, 2005). Shifts in the bacterial community compositions were visualized using a principal coordinate analysis (PCoA) of the pairwise Bray–Curtis dissimilarity matrices of OTUs similarity across the different samples. The beta diversity was determined by additive partitioning approach (Lande, 1996) based on OTUs results.

### 2.6 Statistical analysis

A detrended correspondence analysis (DCA) was used to determine the unimodality or linearity in the chironomid data. A redundancy analysis (RDA) was used to determine those environmental variables that explained most of the variance in the distribution and composition of anammox assemblages.

The CANOCO 4.5 program (Ithaca, NY, USA) was used for all ordinations. The correlations between various variables were computed by Pearson's correlation analysis, which was conducted by SPSS 22.0 for Windows (SPSS Inc., USA). Unless otherwise specified, the level of significance in this study was α = 0.05. The figures were drawn with Origin 9.0.

## 3 RESULTS

### 3.1 Occurrence and abundance of anammox bacteria

**Figure 1:**
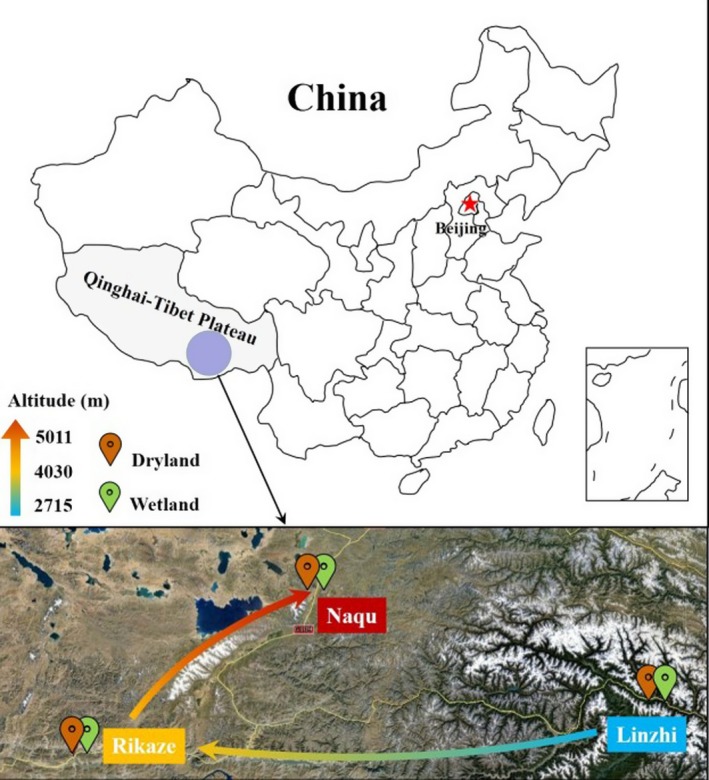
Wetland and dryland soil samples have been sampled in 27 regions with different altitudes from Ya'an (641 m) to Yang bajing (5,033 m) in Qinghai–Tibet Plateau, China. Three representative sampling sites for the analysis of the biogeographical distribution of anammox bacteria, including Linzhi (2,715 m), Rikaze (4,030 m), and Naqu (5,011 m), were labeled on the map from low altitude to high altitude

The 16S rRNA genes of *Planctomycetes* and anammox bacteria at all sampling sites in Qinghai–Tibet Plateau were specifically amplified. Results of sequencing revealed that anammox‐related 16S rRNA genes were only detected in 9 sampling sites of the 50 total sites, and were most closely affiliated to *Candidatus* Brocadia (Table S1). Then soils in wetland and dryland from three regions with different altitudes, including Linzhi (LZ) (3,010 m), Rikaze (RKZ) (3,850 m), and Naqu (NQ) (5,011 m), were further processed to analyze the biogeographical distribution of anammox bacteria in Qinghai–Tibet Plateau (Figure 1).

The physicochemical parameters of the selected soil samples were measured as summarized in Table 1. The NH_4_^+^ concentrations in the wetland soils were higher than in the dryland soils in all of the sites (*p *<* *.05). NO_3_^−^ concentration was higher in the dryland soils than in wetland (*p *<* *.05), being highest in RKZ. NO_2_^−^ concentrations were relatively low at all sites. MC was generally higher in the wetland soils in comparison with the dryland soils regardless of the altitude (*p *<* *.05). The TOM values increased with altitude (*p *<* *.05), but no significant difference was observed between the two types of soils. All sites were slightly alkaline (pH = 7.4–8.6). The surveyed soils in Qinghai–Tibet Plateau had low reactive nitrogen and the concentrations of NH_4_^+^, NO_3_^−^, MC, and TOM varied significantly in Qinghai–Tibet Plateau.

**Table 1:** Physicochemical properties of the selected soil samples along the altitudinal gradient in Qinghai–Tibet Plateau

Site	Linzhi (LZ)		Rikaze (RKZ)		Naqu (NQ)	
Soil type	Wetland	Dryland	Wetland	Dryland	Wetland	Dryland
Altitude (m)	2,715	2,715	4,030	4,030	5,011	5,011
NH_4_^+^ (mg·kg^−1^)	0.89	0.69	4.27	0.77	4.75	2.25
NO_3_^−^ (mg·kg^−1^)	0.68	6.20	0.50	22.11	4.28	6.12
NO_2_^−^ (mg·kg^−1^)	0.04	0.03	0.03	0.03	0.05	0.04
MC (%)	26.05	11.91	21.10	15.37	27.39	17.23
TOM (%)	2.98	2.70	3.94	4.31	4.95	5.25
pH	8.13	7.36	8.07	8.12	8.21	8.55

**Figure 2:**
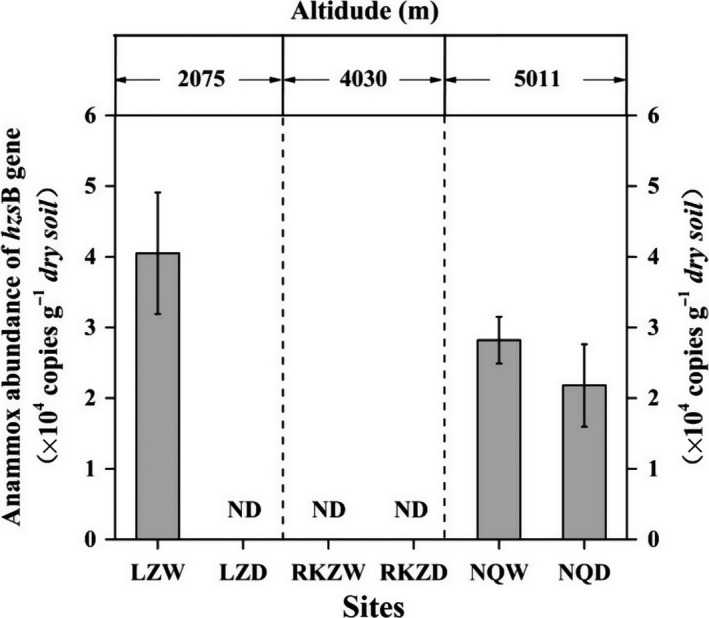
Biogeography distribution of anammox bacterial *hzs*B gene in Qinghai–Tibet Plateau

The abundance of anammox bacteria was estimated using qPCR assays targeting the anammox‐specific *hzs* gene. Results showed that the anammox abundance in the dryland soils of Linzhi (LZD) and also both the soils of Rikaze (RKZW and RKZD) were all below the detection limit (<10^3^ copies·g^−1^) (Figure 2). The highest abundance was observed in the wetland soils of Linzhi (LZW) at a level of 4.05 × 10^5^ copies·g^−1^ dry soil. The abundance of anammox bacteria was relatively higher in the wetland soils at the lower altitude. Spearman's rank correlational analysis also showed that there was no significant correlation between the abundance of anammox bacteria and the investigated soil properties (i.e., soil type, altitude, NH_4_^+^, NO_3_^−^, NO_2_^−^, MC, TOM, and pH) (Table S2).

### 3.2 Diversity of anammox bacteria

A total of 75,959 raw reads generated through high‐throughput pyrosequencing were obtained from the six samples collected from wetland and dryland soils. After quality filtering, a total of 72,099 high‐quality sequences were obtained ranging from 7,541 to 16,502 sequences per sample. Clustering of the sequences at 90% similarity by UCLUST generated operational taxonomic units (OTUs) that ranged from 21 to 41 OTUs depending on the sample (Table 2). Rarefaction curves of alpha diversity reached plateau indicating that the potential true diversity of anammox community had been well captured at the sequencing depth used in this study (Figure S2). This was consistent with the high taxonomic similarity of the OTUs with known representative of anammox species, ranging from 0.99 to even 1.00 at 90% identity cutoff.

**Table 2:** Alpha diversity of anammox bacteria in Qinghai–Tibet Plateau

Site	Linzhi (LZ)		Rikaze (RKZ)		Naqu (NQ)	
Soil type	Wetland	Dryland	Wetland	Dryland	Wetland	Dryland
Altitude (m)	2,715	2,715	4,030	4,030	5,011	5,011
Total reads	16,954	8,135	9,014	8,728	16,588	16,540
Processed reads	16,502	7,541	8,730	8,480	15,271	15,575
OTUs	28.00	34.00	21.00	24.00	33.00	38.00
Chao1	28.50	44.00	21.00	24.75	36.00	41.75
Shannon	0.80	1.55	0.44	0.48	1.35	1.91
Simpson	0.73	0.40	0.86	0.79	0.46	0.30

The Chao1, Shannon, and Simpson indices were calculated to estimate the alpha diversity of anammox bacteria in Qinghai–Tibet Plateau (Table 2). Alpha diversity was a comprehensive norm to represent community richness and diversity. The Chao1 index related positively with community richness, Shannon index was positively correlated with community diversity, while Simpson index correlated negatively with community diversity. The dryland soils showed higher Chao1 and Shannon values than those of the wetland soils, but exhibited a lower Simpson diversity. This indicated that the anammox bacterial community had a higher diversity in the dryland soils than the wetland soils. Furthermore, differences were also found between the three sampling sites regardless of the soil types, showing that the anammox bacterial community had the lowest diversity at the midaltitude site. Similar to the abundances, Spearman's rank correlation did not reveal significant correlations between alpha‐diversity indices and the investigated soil properties (Table S3).

The beta diversity of anammox bacteria in Qinghai–Tibet Plateau was studied using additive partitioning approach (Lande, 1996). Furthermore, the *C*_beta_, representing contribution to gamma diversity, was calculated to quantify and compare the beta diversity among various samples from different points (Crist & Veech, 2006) (Table 3). The calculated results showed that the values of *C*_beta_ were higher (21.80%) in smaller scale (site scale) than larger scale (14.49% for altitude; 3.26% for soil type). This indicated small‐scale environmental heterogeneities played a relatively more dominant role in shaping anammox bacterial community compositions in Qinghai–Tibet Plateau.

**Table 3:** Beta diversity of anammox bacteria in Qinghai–Tibet Plateau

Scale	Area	γ	α	β	*C*_beta_	*C*_beta_ (*M*)
Soil site	LZ	42.00	31.00	11.00	26.19%	21.80%
	LZ1(W)	42.00	28.00	14.00	33.33%	
	LZ2(D)	42.00	34.00	8.00	19.05%	
	RKZW	28.00	22.50	5.50	19.65%	
	RKZ1(W)	28.00	21.00	7.00	25.00%	
	RKZ2(D)	28.00	24.00	4.00	14.29%	
	NQW	46.00	35.50	9.00	19.57%	
	NQ1(W)	46.00	33.00	13.00	28.26%	
	NQ2(D)	46.00	38.00	5.00	10.87%	
Altitude	2,715 m	46.00	42.00	4.00	8.70%	14.49%
	4,030 m	46.00	28.00	16.00	34.78%	
	5,011 m	46.00	46.00	0.00	0.00%	
Soil type	Wetland	46.00	44.00	2.00	4.35%	3.26%
	Dryland	46.00	45.00	1.00	2.17%	

All of the indices were calculated based on OTUs results; γ = the total species richness in a certain region; α *=* the average species richness in sampling points in the region; β = γ *−* α; *C*_beta_ = β*/*γ* *× 100%.

### 3.3 Community composition of anammox bacteria

**Figure 3:**
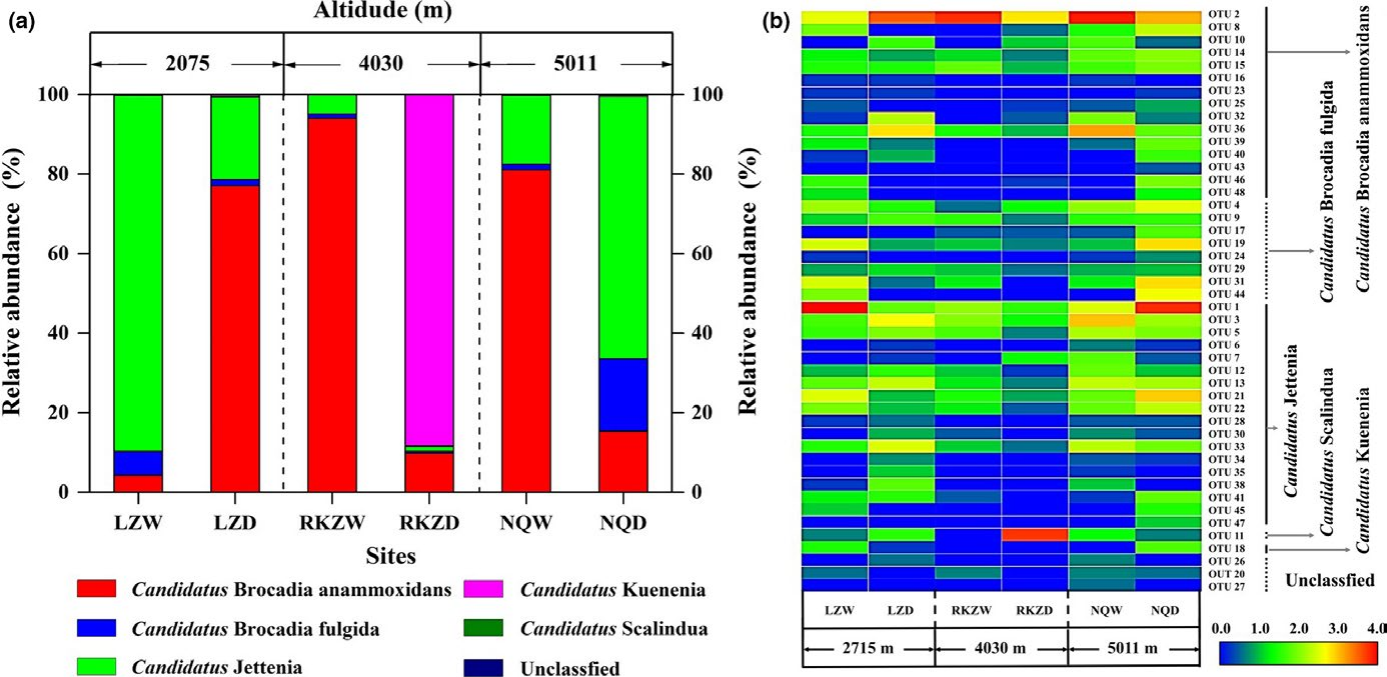
The community compositions of anammox bacteria (a) and a heatmap of anammox bacterial community compositions along the altitude in different soil types of Qinghai–Tibet Plateau (b)

A local BLAST analysis of the amplified *hzs*B protein sequences was performed to assign identity of the sequences. Five protein sequences were used as references for BLAST (Table S6). Results showed that *Candidatus* Brocadia anammoxidans (46.9%) and *Candidatus* Jettenia (33.3%) were the dominant groups, followed by *Candidatus* Brocadia fulgida (14.9%) and *Candidatus* Scalindua (0.1%) at the sampling sites based on mean relative abundance (Figure 3a). The community composition of anammox bacteria also differed between the wetland and dryland soils. A detailed comparison of the anammox bacterial composition was presented as a heatmap (OTUs level) in Figure 3b. Specifically, *Candidatus* Brocadia anammoxidans were higher in the wetland soils of RKZ (94.1%) and NQ (81.0%) than that in the dryland soils but were significantly lower in the wetland soils of LZ (4.3%). *Candidatus* Jettenia had higher abundance in the wetland soils of LZ (89.5%) and RKZ (4.9%) and in the dryland soils of NQ (66.1%) compared to the other kinds of soils. In particular, the overall abundance level of *Candidatus* Jettenia at the site of RKZ was lower in comparison to that at the sites of LZ and NQ. As for *Candidatus* Brocadia fulgida, it showed low abundance at all sites and only had a relatively higher abundance in the wetland soils of LZ (5.9%) and in the dryland soils of NQ (18.1%). Moreover, *Candidatus* Kuenenia was mainly detected in the dryland soils of RKZ (88.5%) and was rarely observed at the other sites (<0.5%). *Candidatus* Scalindua was only detected in the soils of NQ and the dry soils of LZ, accounting for a small fraction (<0.5%).

**Figure 4:**
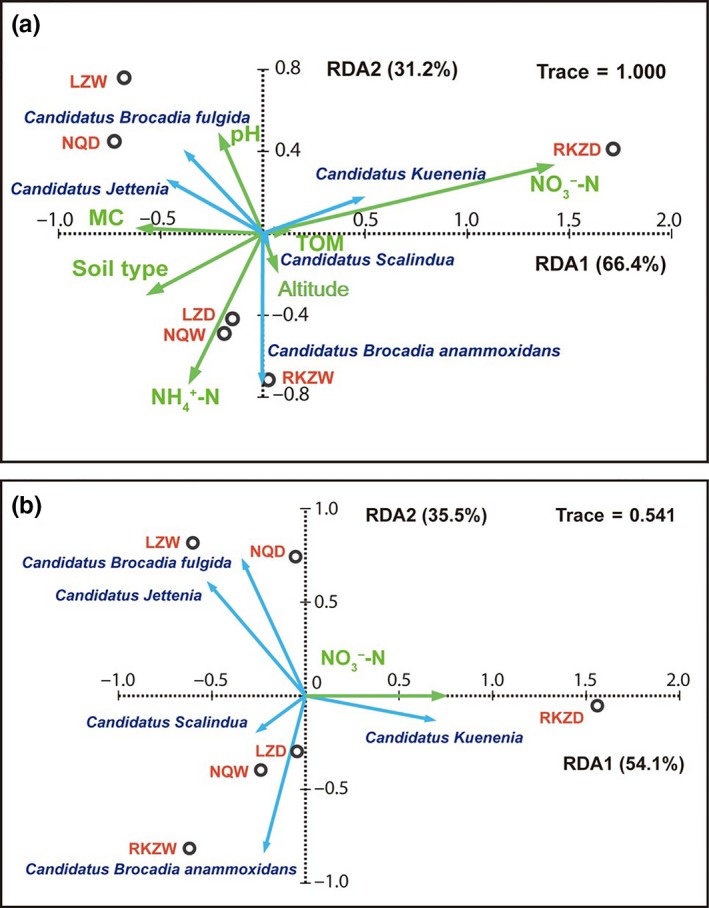
RDA ordination diagram of anammox bacterial community composition associated with environmental variable including soil type, altitude, NH_4_^+^, NO_3_^−^, MC, TOM, and pH (a), and the next‐step RDA with NO_3_^−^ in soils (b). The percentage indicated the interpretation of axis to the diversification of anammox bacterial community

Spearman's rank correlation analysis between the anammox community composition and the investigated soil properties revealed that the relative abundance of *Candidatus* Kuenenia of anammox bacteria was significantly correlated with the changes in NO_3_^−^ (*p *<* *0.05) (Table 4). In order to further understand this relationship, multivariate statistical analysis was performed in Canoco 4.5. First, detrended correspondence analysis (DCA) was carried out to determine the unimodality of the taxonomic abundance. The gradient lengths for the first four axes were 1.529, 0.469, 0.807, and 0.860, indicating that anammox community had a linear distribution (gradient length should be higher than 3 to indicate unimodality). Afterward, a forward selection was used to determine the variables that significantly contribute to the observed patterns (Table S4). Based on the results, the variance explained by the different parameters, from highest to lowest, was NO_3_^−^ (54.1%) > NH_4_^+^ (16.7%) > soil type (10.3%) > MC (9.4%) > pH (7.6%) > altitude (1.7%) > TOM (1.3%). However, only NO_3_^−^ had a significant influence on the anammox composition (*p *<* *0.05). Next‐step redundancy analysis (RDA) and Monte Carlo test (999 permutations) (Table S5) were further conducted with NO_3_^−^. The data as shown in Figure 4 indicated that the first RDA axes explained 54.1% of the variance in the anammox assemblages and the species–environmental correlation at the first RDA axes was 91.5%.

**Table 4:** Spearman's correlation analysis between relative abundance of anammox bacteria and soil properties (*n* = 6)

Anammox bacterial taxa	Soil type	Altitude	NH_4_^+^	NO_3_^−^	MC	TOM	pH
*Candidatus* Brocadia anammoxidans	0.293	0.359	0.543	−0.371	0.143	0.086	−0.257
*Candidatus* Brocadia fulgida	−0.098	0.000	−0.029	−0.143	0.086	0.086	0.486
*Candidatus* Jettenia	0.098	−0.239	−0.086	−0.257	0.200	−0.143	0.371
*Candidatus* Kuenenia	−0.683	0.000	−0.543	0.943**	−0.543	0.029	−0.143
*Candidatus* Scalindua	−0.311	−0.127	−0.273	0.030	−0.152	0.030	0.395

**Correlation is significant at the 0.01 level (2‐tailed); * Correlation is significant at the 0.05 level (2‐tailed).

## 4 DISCUSSION

The present study is the first report about the occurrence, diversity, community composition, and abundance of anammox bacteria in two types of soils (wetland and dryland soils) along the altitudinal gradient of an alpine ecosystem (Qinghai–Tibet Plateau). The findings in this study would contribute further understanding on the anammox process occurring in the plateau, and indicate that small‐scale environmental heterogeneities are important in shaping the community composition and abundance of anammox bacteria.

Knowledge on the diversity of bacteria in ecosystems is key information needed for understanding the underlying mechanisms of global nitrogen cycle (Philippot et al., 2013; Taroncher‐Oldenburg, Griner, Francis, & Ward, 2003). Our study illustrated that the anammox bacteria had lower diversity in wetland than dryland soils, indicating distinctive spatial heterogeneity in alpine ecosystems between wetland and dryland. To date, the diversity of anammox bacteria has been studied in various ecosystems. In aquatic ecosystems, widespread occurrence but low diversity of anammox were shown in marine ecosystems (Schmid et al., 2007). So far, the available anammox 16S rRNA sequences from marine (e.g., Black Sea and the Benguela OMZ, Namibia) and estuarine environments (e.g., Randers Fjord, Denmark) were all related to *Candidatus* Scalindua. Anammox diversity in freshwater ecosystem was higher than in marine, for example, the 16S rRNA genes in the sediments of Xinyi River were closely related to *Candidatus* Brocadia anammoxidans (Zhang et al., 2007) and in some freshwater extreme environments, most *hzs*B gene sequences were closely affiliated to *Candidatus* Kuenenia (Zhu et al., 2015). Anammox diversity in terrestrial system was higher in soil than in freshwater environments, for example, in paddy soils, high alpha diversity (Shannon index = 1.84–2.71) was found and anammox bacteria is related to *Candidatus* Brocadia, *Candidatus* Kuenenia, and two novel unidentified clusters (Yang et al., 2015). In vegetable soils, the Shannon index was also high (2.04–2.59) and three different genera of anammox bacteria are detected, including *Candidatus* Kuenenia, *Candidatus* Brocadia, and *Candidatus* Jettenia (Shen, Wu, Liu, & Li, 2017). The above discussions indicated in natural ecosystems, the highest diversity of anammox bacteria occurred in terrestrial systems, followed by freshwater systems and marine systems, which was in accordance with our finding that anammox bacteria had higher diversity in dryland than wetland of alpine system.

High‐throughput pyrosequencing analysis of the community composition of anammox bacteria in alpine ecosystem showed that the dominant anammox taxa in the studied regions were *Candidatus* Brocadia, Jettenia, and Kuenenia. In contrast to marine pelagic waters where *Candidatus* Scalindua dominates anammox guilds, Kuenenia and Brocadia appear to be the most common representatives in terrestrial environments (Humbert et al., 2010). In the activated sludge wastewater treatment plants, *Candidatus* Brocadia or *Candidatus* Jettenia are the dominant species in the anammox community (Suto et al., 2017; Wang, Peng, Ma, Wang, & Zhu, 2015). This observation suggests environmental selection of anammox bacteria in natural and engineered ecosystems. However, in the present study, it was not found that the anammox bacteria showed a variation with altitude at large scale, and anammox bacteria community showed a very strong heterogeneity between different points in each altitude. This indicates small‐scale environmental heterogeneities are important in shaping the community composition and abundance of anammox bacteria.

Certain habitat‐specific studies have shown that anammox bacterial distribution is affected by different environmental factors, for example, temperature (Hou et al., 2013), and available nitrite and ammonium concentrations (Li & Gu, 2013). A recent study using global data ordination demonstrated that salinity is one of the key factors driving the biogeography of the anammox bacteria (Sonthiphand et al.,^2014^). The physiological properties of anammox bacteria, including the specific growth rate (μ_max_), affinity for ammonia and nitrite (*K*_*s*_), optimum growth temperature, and pH should be attributed to this selection. For instance, anammox bacteria *Candidatus* Brocadia sinica adapts better to various ecosystems because of their lower affinity for ammonia and nitrite, higher tolerance to O_2_, and higher growth rate (Oshiki, Shimokawa, Fujii, Satoh, & Okabe, 2011). A shift from a *Candidatus* Brocadia dominated community to a *Candidatus* Kuenenia was observed in fluctuating nitrite concentrations due to differences in affinity for NO_2_^−^ (Wr et al., 2008). In this study, RDA and Spearman's correlation analysis indicated that the community composition of anammox bacteria significantly correlated with the concentrations of NO_3_^−^. Particularly, NO_3_^−^ was correlated well with the relative abundance of *Candidatus* Kuenenia (*p *<* *.05). However, the role of other unidentified ecological parameters cannot be ruled out (Shehzad et al., 2016).

Quantitative PCR results showed that the abundance of anammox bacteria in Qinghai–Tibet Plateau was relatively low (2.18 × 10^4^–4.05 × 10^5^ copies·g^−1^ dry soil) in comparison to those in other natural or seminatural ecosystems, such as the marsh sediments of Yangtze Estuary (2.63 × 10^6^–1.56 × 10^7^ copies·g^−1^ dry soil), the riparian sediments of Pearl River Estuary (1.30 × 10^4^–2.00 × 10^9^ copies·g^−1^ dry soil), and constructed wetlands (up to 3.38 × 10^7^ copies·g^−1^ dry soil) (Hou et al., 2013; Zhu et al., 2011). Interestingly, the anammox abundance in RKZ could not be determined by qPCR but were detected by PCR, which may be explained by the different detection limits between qPCR (~10^3^ copies·g^−1^ dry soil) and nested PCR (~10^1^ copies·g^−1^ dry soil). The reactive nitrogen was the fundamental factor maintaining the stability of the anammox reactions (Dalsgaard & Bo, 2002; Jetten et al., 2009). The Qinghai–Tibet Plateau received low load of reactive nitrogen. In addition, the surveyed regions were perennially in low temperature. In the year we conducted the study (2015), the temperature was −12°C to 15°C in 8 months of the year (http://www.tianqi.com/), while anammox bacteria were more abundant at higher temperatures, that is, their optimum temperature in temperate shelf sediments was 15°C (Dalsgaard et al., 2005). As a result, the growth of anammox bacteria was limited in such adverse conditions causing the observed low abundance of anammox bacteria in Qinghai–Tibet Plateau.

In this study, RDA and Spearman's correlation analysis indicated that the NO_3_^−^ was the most important environmental factor influencing the community composition of anammox bacteria in Qinghai–Tibetan Plateau. In natural ecosystems, NO_3_^−^ mainly came from microbial nitrification driven by ammonium oxidizers including ammonia‐oxidizing archaea (AOA) and bacteria (AOB). The results from Xie et al. (2014) and Tai et al. (2014) indicated that both AOA and AOB mediated NO_3_^−^ production, of which the archaeal nitrification dominated over bacteria in nitrification process. This suggested that there may be a good symbiotic relationship between AOA and anammox bacteria in Qinghai–Tibetan Plateau.

## CONFLICT OF INTEREST

The authors declare no conflict of interest.
