# Supplementary material for: High‐throughput analysis of anammox bacteria in wetland and dryland soils along the altitudinal gradient in Qinghai–Tibet Plateau
Source: Microbiologyopen. 2017 Dec 29;7(2):e00556. doi: 10.1002/mbo3.556 (PMC5911990; doi:10.1002/mbo3.556)
Supplement: Supplementary file 1 [file MBO3-7-na-s001.docx]

**Supplementary Information**


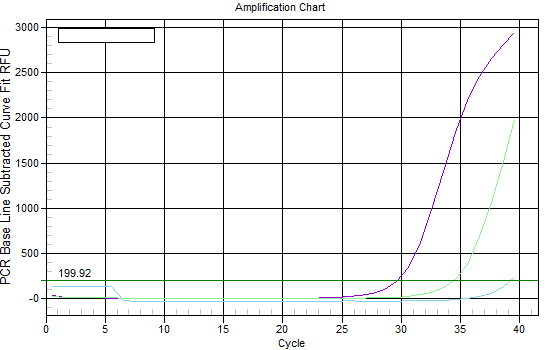


**negative control**

**Figure S1** PCR Amp/Cycle Chart of standard curve


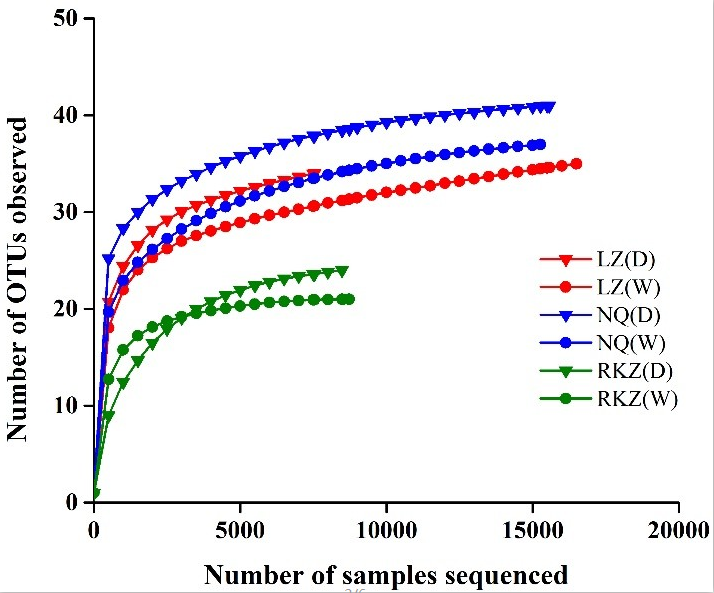


**Figure S2** Rarefaction analysis of anammox bacterial communities

**Table S1** Background of sampling sites in Qinghai-Tibet Plateau

| **Sites** | **Coordinate** | **Altitude (m)** | **Location** | **Soil types** | **Anammox** |
| --- | --- | --- | --- | --- | --- |
| Ya'an | 29°59′35″N, 103°00′22″E | 641 | Farmland | Dyrland | Negative |
| Luding | 29°55′44″N, 102°13′48″E | 1330 | Farmland | Dyrland | Negative |
| Tongmai | 30°06′11″N, 95°04′47″E | 2070 | Cornfield | Dyrland | Negative |
| Kangding | 29°59'35''N, 101°53'41''E | 2395 | Farmland | Dyrland | Negative |
| Batang | 30°01′12″N, 99°05′60″E | 2580 | Farmland | Dyrland | Negative |
| **Bomi** | **29°51'46''N, 95°46'15''E** | **2725** | **Wetland** | **Wetland** | **Positive** |
| **Bomi** | **29°51'46''N, 95°46'15''E** | **2725** | **Wheat field** | **Dyrland** | **Positive** |
| Bayi | 29°38′24″N, 94°21′37″E | 2930 | Grassland | Dyrland | Negative |
| Bayi | 29°38′24″N, 94°21′37″E | 2930 | Rape field | Dyrland | Negative |
| Bayi | 29°38′24″N, 94°21′37″E | 2930 | Wetland | Wetland | Negative |
| Linzhi | 29°43′48″N, 94°20′60″E | 3010 | River | Wetland | Negative |
| Linzhi | 29°43′48″N, 94°20′60″E | 3010 | Grassland | Dyrland | Negative |
| Basu | 30°03′36″N, 96°55′12″E | 3280 | Cornfield | Dyrland | Negative |
| Lulang | 29°56′38″N, 94°47′57″E | 3285 | Grassland | Dyrland | Negative |
| Lulang | 29°56′38″N, 94°47′57″E | 3285 | Swamp | Wetland | Negative |
| Gongbujiangda | 29°53′24″N, 93°15′00″E | 3330 | Forest | Dyrland | Negative |
| Gongbujiangda | 29°53′24″N, 93°15′00″E | 3330 | Swamp | Wetland | Negative |
| Qushui | 29°21′14″N, 90°44′31″E | 3598 | River | Wetland | Negative |
| Qushui | 29°21′14″N, 90°44′31″E | 3598 | Grassland | Dyrland | Negative |
| Xinduqiao | 30°31'26''N, 101°31'29''E | 3630 | Swamp | Wetland | Negative |
| Lasa | 29°38′49″N, 91°08′24″E | 3650 | Grassland | Dyrland | Negative |
| Lasa | 29°38′49″N, 91°08′24″E | 3650 | Forest | Dyrland | Negative |
| Lasa | 29°38′49″N, 91°08′24″E | 3650 | Wetland | Wetland | Negative |
| Mozhugongka | 29°50′10″N, 91°43′48″E | 3840 | Wheat field | Dyrland | Negative |
| Rikaze | 29°16′02″N, 88°52′51″E | 3850 | River | Wetland | Negative |
| Rikaze | 29°16′02″N,88°52′51″E | 3850 | Vegetable field | Dyrland | Negative |
| Rikaze | 29°16′02″N,88°52′51″E | 3850 | Forest | Dyrland | Negative |
| Mangkang | 29°40′48″N,98°35′24″E | 3875 | Grassland | Dyrland | Negative |
| **Mangkang** | **29°40′48″N, 98°35′24″E** | **3875** | **River** | **Wetland** | **Positive** |
| Zuogong | 29°42′36″N,97°49′48″E | 3877 | Stream | Wetland | Negative |
| Zuogong | 29°42′36″N,97°49′48″E | 3877 | Grassland | Dyrland | Negative |
| Zuogong | 29°42′36″N,97°49′48″E | 3877 | Wheat field | Dyrland | Negative |
| Renbu | 29°13′52″N,89°50′31″E | 3894 | River | Wetland | Negative |
| Renbu | 29°13′52″N,89°50′31″E | 3894 | Potato field | Dyrland | Negative |
| Ranwu | 29°30'18''N,96°41'18''E | 3960 | Wetland | Wetland | Negative |
| Ranwu | 29°30'18''N,96°41'18''E | 3960 | Lake | Wetland | Negative |
| Ranwu | 29°30'18''N,96°41'18''E | 3960 | Wheat field | Dyrland | Negative |
| Litang | 29°25'29''N,100°17'13''E | 4014 | Wetland | Wetland | Negative |
| **Lazi** | **29°05′13″N, 87°37′48″E** | **4030** | **Stream** | **Wetland** | **Positive** |
| **Lazi** | **29°05′13″N, 87°37′48″E** | **4030** | **Wheat field** | **Dyrland** | **Positive** |
| **Naqu** | **31° 4'17"N, 92°22'2"E** | **5011** | **River** | **Wetland** | **Positive** |
| **Naqu** | **31° 4'17"N, 92°22'2"E** | **5011** | **Grassland** | **Dyrland** | **Positive** |
| Dangxiong | 30°25'40"N,91° 5'50"E | 5021 | Grassland | Dyrland | Negative |
| Dangxiong | 30°25'40"N,91° 5'50"E | 5021 | Wetland | Wetland | Negative |
| Bangda | 30°15'20"N,97°10'2"E | 5024 | Wheat field | Dyrland | Negative |
| Bangda | 30°15'20"N,97°10'2"E | 5024 | Wetland | Wetland | Negative |
| Gulu | 30°35'456"N, 91° 7'14"E | 5027 | Stream | Wetland | Negative |
| **Gulu** | **30°35'456"N, 91° 7'14"E** | **5027** | **Grassland** | **Dyrland** | **Positive** |
| **Yangbajing** | **30° 6'50''N, 90°34'24"E** | **5033** | **Grassland** | **Dyrland** | **Positive** |
| Yangbajing | 30° 6'50''N, 90°34'24"E | 5033 | Stream | Wetland | Negative |

**Table S2** Spearman correlation analysis between anammox bacterial abundance and soil properties (*n*=6)

| Abundance | Soil type | Altitude | NH_4_^+^ | NO_3_^-^ | MC | TOM | pH |
| --- | --- | --- | --- | --- | --- | --- | --- |
| *Anammox* | 0.518 | 0.127 | 0.395 | -0.395 | 0.759 | 0.213 | 0.698 |

** *P* < 0.01, **P* < 0.05.

**Table S3** Spearman correlation analysis between α-diversity indexes of anammox bacteria and soil properties (n=6)

| α-diversity index | Soil type | Altitude | NH_4_^+^ | NO_3_^-^ | MC | TOM | pH |
| --- | --- | --- | --- | --- | --- | --- | --- |
| Chao 1 | -0.488 | 0.000 | -0.314 | 0.429 | -0.314 | -0.029 | 0.143 |
| Shannon | -0.488 | 0.239 | -0.143 | 0.371 | -0.200 | 0.257 | 0.429 |
| Simpsom | 0.488 | -0.239 | 0.143 | -0.371 | 0.200 | -0.257 | -0.429 |

** *P* < 0.01, **P* < 0.05.

**Table S4** Forward test of anammox bacterial composition and soil properties (n=6)

|  | Soil type | Altitude | NH_4_^+^ | NO_3_^-^ | MC | TOM | pH |
| --- | --- | --- | --- | --- | --- | --- | --- |
| Variables | 0.103 | 0.017 | 0.167 | 0.541 | 0.094 | 0.013 | 0.076 |
| F | 0.459 | 0.071 | 0.803 | 4.706 | 0.417 | 0.052 | 0.329 |
| *p* | 0.694 | 0.900 | 0.475 | 0.007 | 0.716 | 0.964 | 0.640 |

**Table S5** Monte Carlo test (999 permutation) between anammox bacterial composition and NO_3_^-^ in soils (*n*=6)

| Axes | 1 | 2 | 3 | 4 | Total variance |
| --- | --- | --- | --- | --- | --- |
| Eigenvalues | 0.541 | 0.355 | 0.084 | 0.020 | 1.000 |
| Species-environmental correlation | 0.915 | 0.000 | 0.000 | 0.000 |  |
| Cumulative percentage variance  of species data  of species-environment relation |  |  |  |  |  |
|  | 54.1 | 89.6 | 98.0 | 100.0 |  |
|  | 100.0 | 0.0 | 0.0 | 0.0 |  |
| Sum of all eigenvalues  Sum of all canonical eigenvalues |  |  |  |  | 1.000 |
|  |  |  |  |  | 0.541 |

**Table S6** Reference protein sequences for BLAST

>AFA41965.1Candidatus_Brocadia_sp.a

GWGSWKNVKYIRGGRYLPPFRHEGFTGHPDEIVGATSSLDRVCGRDPGFVFRSENFSPERLESLICYIRALEFTGSPFRNADGSLTEAAKRGEKLFNDPAVGCAECHPGDAMDPKALFSDTQTHDVGT

>AFA41966.1Candidatus_Brocadia_sp.f

GWGSWKNTKYIRGGRYLPPFRHEGFTGHPDEIVGATSSLDRVCGRDPGSVFRSENFSPLRLEALICYIRALEFTGSPFRNPDGSLTDAQKRGEKIFNDPNVGCVECHPGDASDPKALFSDAQTHDVGT

>AFA41967.1Candidatus_Jettenia_sp.J

GWGSWKNTKYIRGGRYLPPFRHEGFTGHPDEIVGATSSLDRVCGRDPGFVFRSENFSPERLESIICYIRSLEFTGSPFRNADGSLTEAQKRGEKLFNDPKVGCVECHPGESSDPKALYSDAQTHDVGT

>AFA41968.1Candidatus_Kuenenia_sp.K

GWGSWKNTKYIRGGRYLPPFRHEGFTGHPDEIVGATSSLDRVCGRDSGFVFRSENFSPMRLEALICYIRALEFTGSPFRNADGSLTEAQKRGQKIFEDPKVGCLECHPGDPMDPRALFSDAQTHDVGT

>AFA41969.1Candidatus_Scalindua_sp.S

GWGSWKNTKYIRGGRYLPPFRHEGFTGHPDEIVGAASSIDRVCGRDPGFVFRSENFSPERLEALIAYIRSLEFTGSPFRKEDGSLTEAQKRGWKIFSDPKVGCMECHPGDPKNPRALFSDAQTHDVGT
